# Stromal Cell Induction of Regulatory Dendritic Cells

**DOI:** 10.3389/fimmu.2012.00262

**Published:** 2012-08-21

**Authors:** Benjamin M. J. Owens, Paul M. Kaye

**Affiliations:** ^1^Centre for Immunology and Infection, Hull York Medical School and Department of Biology, University of YorkYork, UK

**Keywords:** stromal cells, dendritic cells, Immune regulation, IL-10, inflammation, infection

## Abstract

Dendritic cells (DCs) are specialized antigen presenting cells of bone marrow origin that can exist in tissues in either an immature or mature state. DCs have a myriad of roles in immunity and tolerance induction, but are perhaps best known for their role in the activation and differentiation of naïve T cells at the onset of an acquired immune response. Over the past decade, a body of literature has developed that suggests that DCs, as well as many other myeloid cell populations, are also capable of exerting “regulatory” effects on T cell responses. However, relatively little is known regarding the mechanisms by which such regulatory myeloid cells arise *in vivo*. In this mini-review, we first define the characteristics of “regulatory” DCs (rDCs) and then focus on the contribution of non-hematopoietic stromal cells to their generation within specific tissue microenvironments. We also highlight areas of research that warrant future attention, arguing for a focusing of efforts toward a better understanding of the features of stromal cell populations that enable the induction of rDCs. Finally, we discuss how an understanding of stromal cell-myeloid cell interactions may lead to new therapeutic strategies for cancer, autoimmunity, and infectious disease.

## Introduction

Dendritic cells (DCs) lie at the interface of innate and adaptive immunity, playing a critical role in the initiation of effective T cell-mediated immune responses. Paradoxically, DCs also have the potential to exert powerful negative regulatory effects on the immune system (Steinman et al., 2003). This potential for dampening immunity has spawned great interest in the context of cell-based therapeutic intervention in a variety of autoimmune and inflammatory contexts (Kalantari et al., 2011). Alongside several populations of conventional CD11c^hi^ DCs (cDCs), there are many other myeloid cell populations capable of antigen presentation and exerting both positive and negative effects on T cell responses. This has given rise to a literature that contains a significant level of confusion. That many of these myeloid cells also share phenotypic characteristics has only compounded the problem. For example, the assignment of cells as DCs based solely on CD11c expression has not always been helpful (Drutman et al., 2012). The relationship between cDCs and other myeloid cells is further complicated by the existence of convergent differentiation pathways, particularly under inflammatory conditions (Geissmann et al., 2010). Nevertheless, recent data strongly support the concept that cDCs belong to a distinct immune cell lineage (Meredith et al., 2012; Satpathy et al., 2012). The identification of key lineage-related transcription factors should allow rapid progress in determining the relationships between cDCs and other myeloid cell populations and add some clarity to studies of the regulatory properties of these cells.

**Figure 1:**
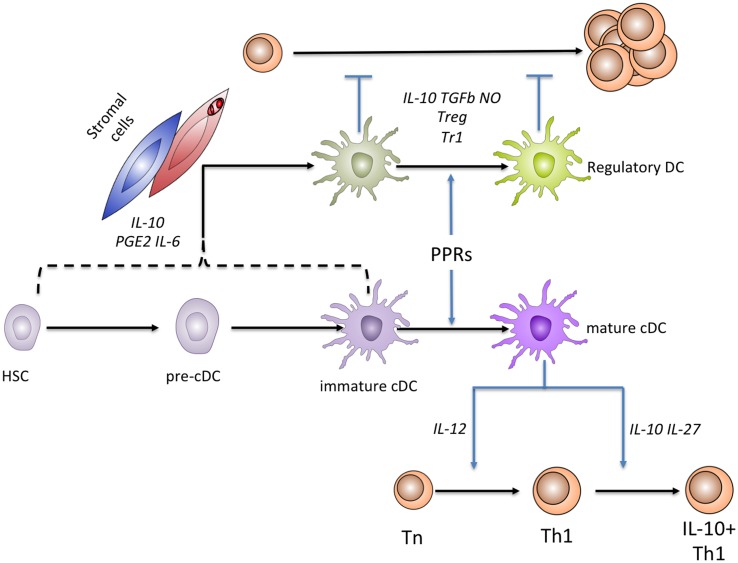
**Relationships between rDCs and cDCs**. cDCs are known to originate from a hematopoietic stem cell precursor, through a sequence of events (not shown) that culminates in production of tissue precursor cells (pre-cDCs). In tissue, cDCs exist as immature cells, but functionally mature in response to microbial sensing mediated through various pattern recognition receptors (PRRs). Mature cDCs have an enhanced capacity to prime naïve T cells and induce their differentiation (shown here for Th1 cells). Mature cDCs may also help terminate excessive T cell responses by inducing a “self regulatory” phenotype, e.g., through the induction of IL-10 production by Th1 cells. Under steady state conditions, stromal cells (blue) are able to induce the generation of IL-10 and PGE_2_-producing rDCs from c-kit^+^ progenitors by production of PGE_2_, IL-6, and IL-10, as well as direct interactions mediated by Fibronectin. During the chronic inflammation associated with *L. donovani* infection, IL-10-producing rDC generation is enhanced by the production of CCL8 and CXCL12 by splenic stromal cells that in many cases are directly parasitized (red). The stage(s) of development at which cDCs or their precursors are able to differentiate into regulatory DCs is not yet defined. Functionally, regulatory DCs are best defined by their capacity to inhibit the proliferative capacity of T cells. rDCs employ multiple mechanisms to suppress naïve T cell proliferation, including the direct production of IL-10 and the induction of FoxP3^+^ nTreg and IL-10^+^ Tr1 Treg populations.

Whilst the regulatory potential of cDCs *in vivo* has long been appreciated (Hawiger et al., 2001), consensus regarding the phenotypic features of cDCs and myeloid cells with regulatory properties has been hard to reach. Historically, “immature” cDCs were classed as those that had yet to receive a pathogen-derived signal and existed in the tissues in a state of readiness for antigen presentation, with high levels of endocytosis to facilitate antigen capture and large intracellular pools of MHCII. Upon pathogen recognition by TLRs or other pattern recognition receptors, cDCs “mature” and in so doing, shut down endocytosis in favor of MHCII-peptide display, heightened expression of co-stimulatory molecules, and the secretion of cytokines that direct naïve T cell differentiation. Early literature suggested that immature cDCs may also be endowed with regulatory function, although this may represent an oversimplification (Kleindienst et al., 2005). Conversely, all subsets of splenic cDC have recently been shown to be capable of producing the regulatory cytokine IL-10, even after TLR induced maturation (Maroof and Kaye, 2008; Owens et al., 2012). In the context of chronic *Leishmania donovani* infection, IL-10-producing cDCs are capable of antigen presentation and the induction of naïve T cell proliferation *in vitro* (Owens et al., 2012), making them functionally distinct from rDCs as we define below. Against this background, where pleiotropic function characterizes cDCs, it becomes pertinent to ask whether there are distinct populations of DCs (regulatory DCs; rDCs) in which regulatory function is hardwired, and how stromal cell populations can contribute to their generation (Figure 1). The remainder of this review will focus on addressing this question.

## Regulatory DCs: Characterization and Function

Amongst cytokines, IL-10 has become synonymous with the concept of regulation, yet as discussed above cDCs under appropriate circumstances are quite capable of producing this cytokine. Hence, IL-10 alone could not be a sufficient criterion by which to distinguish rDCs. Although there is evidence of a rDC population with functions that are distinct from cDCs, there is currently nothing known as to the extent of plasticity within this group of myeloid cells. In particular it is not yet clear whether rDCs represent a terminally differentiated DC phenotype, or a transient functional state reflecting phenotypic changes of myeloid cells in distinct tissue microenvironments. Despite such ambiguity in the nature of rDCs, some of the strongest evidence in support of the existence of this population has come from the study of how fibroblasts and endothelial cells impact on DC development from hematopoietic stem cells or committed myeloid progenitors.

Stromal cell induction of rDC differentiation can occur in multiple tissues even in the absence of pathogen recognition and inflammation, suggesting that this is a normal homeostatic process. To date, stromal cell-induced rDCs have been reported in murine spleen (Svensson et al., 2004; Zhang et al., 2004; Tang et al., 2006; Nguyen Hoang et al., 2010; Xu et al., 2012), liver (Xia et al., 2008), kidney (Huang et al., 2009), lung (Li et al., 2008), and tumor tissue (Liu et al., 2009). Despite their divergent tissue localization, the majority of studies reporting stromal cell-induced rDCs have characterized them as populations of CD11c^lo^ MHCII^lo/int^ CD11b^+^cells, based on surface protein expression assessed by flow cytometry. Splenic rDCs have also been reported to express CD45RB (Wakkach et al., 2003; Svensson et al., 2004), although the functional significance of this is not known. Expression of co-stimulatory molecules such as CD40, CD80, and CD86 is generally lower on rDCs than cDCs, suggesting an impaired capacity to deliver activatory signals to naïve T cell populations, although high co-stimulatory molecule expression (particularly CD80) by rDCs has been reported in some contexts (reviewed by Svensson and Kaye, 2006).

Several other characteristics of stromal cell-induced rDCs allow for their more rigorous identification. Multiple studies have reported that rDCs are major producers of the anti-inflammatory cytokine IL-10, including those from spleen (Svensson et al., 2004; Zhang et al., 2004; Tang et al., 2006), liver (Xia et al., 2008), kidney (Huang et al., 2009), and lung (Li et al., 2008), suggesting that IL-10 production is a conserved feature of rDCs, irrespective of tissue localization, or origin. This preferential IL-10 production can be driven by TLR triggering, and at least partially relies on ERK signaling (Qian et al., 2006). However, conventional CD11c^hi^MHCII^hi^cDCs are also capable of abundant IL-10 production (Saraiva and O’Garra, 2010), a process particularly pronounced during chronic parasitic infection (Maroof and Kaye, 2008; Owens et al., 2012) indicating that IL-10 production is not unique to rDCs.

Distinctive functional properties of rDCs provide perhaps the best method for their identification, as rDCs utilize multiple pathways in order to exert their immune regulatory function. Several reports suggest that rDCs are capable of modulating T cell proliferation *in vitro* and *in vivo*, with splenic stromal-induced rDCs suppressing proliferation via IL-10, TGFβ, and/or nitric oxide (NO) production (Svensson et al., 2004; Zhang et al., 2004; Tang et al., 2006), and pulmonary stromal-induced rDCs inhibiting T cell proliferation via production of Prostaglandin-E_2_ (PGE_2_; Li et al., 2008). Importantly this capacity for stromal-induced rDCs to suppress T cell proliferation does not appear to be dependent on the presence of stromal cells within the assays, as stromal-induced rDCs are capable of suppressing T cell proliferation during *in vitro* co-culture solely with T cells, as well as after adoptive transfer *in vivo*. This therefore indicates a regulatory process distinct from that recently reported for lymph node (LN) fibroblastic reticular cells (FRCs), whereby nitric oxide produced by LN FRCs regulates T cell proliferation via direct effects on T cells, in addition to modulating cDC function (Khan et al., 2011; Lukacs-Kornek et al., 2011; Siegert et al., 2011). Stroma-induced rDCs within tumors are able to utilize an alternative mechanism involving the direct production of Arginase-1 (Liu et al., 2009). This results in the metabolism of l-Arginine and consequent suppression of T cell proliferation *in vitro* and *in vivo*. In contrast, splenic stroma-induced rDCs specifically recruit CXCR3^+^ Th1 cells to more efficiently suppress their proliferation, via the IFNα/β-dependent production of IP-10 after TLR triggering (Qian et al., 2007). More recent evidence has suggested that splenic rDCs are also able to directly induce apoptosis of activated CD4^+^ T cells by a process involving NO, Fas-Ligand, and IFNγ (Xu et al., 2012), a mechanism also reported to occur in the liver (Xia et al., 2008). This capacity for direct suppression of CD4^+^ T cell proliferation, even after TLR induced “maturation,” is a cardinal feature of rDCs that allows for them to be distinguished from cDCs.

In addition to their direct modulatory effects on activated T cells, rDCs can also employ indirect immune regulatory mechanisms, in many cases by the induction of specialized populations of regulatory T cells (Treg). Splenic stroma-induced rDCs directly induce IL-10-producing Tr1 Treg *in vitro* (Svensson et al., 2004), whereas rDCs induced by pulmonary stromal cells can induce populations of Foxp3^+^ natural Treg (Li et al., 2008). Whether the differential induction of Treg populations from rDCs induced by stromal cells of distinct tissues reflects the polarization of rDC subsets is currently unclear. It will be important to define stroma-induced rDCs from distinct tissues in more detail to elucidate whether further functional subsets exist, in addition to the cues that drive their differentiation. However, Treg induction by rDCs does not always contribute to their regulatory potential (Tang et al., 2006) and indeed rDCs have been reported to activate NK cells in some circumstances (Qian et al., 2006). These divergent functions suggest that although rDCs are capable of suppressing the cellular components of an active immune response, in different contexts rDCs may also act in an immune stimulatory fashion.

Taking these multiple parameters into consideration, a “minimal” definition of stromal-induced rDCs can be reached as CD11c^lo/int^ MHCII^lo/int^ CD11b^+^ myeloid cells, capable of IL-10 production, the direct suppression of CD4^+^ T cell proliferation and (in some cases) the induction of Treg. Although several other functional features can also contribute to the identification or characterization of rDCs, these are either tissue-context dependent, or have not yet been assessed in all stromal-induced rDC populations.

## Specific Contexts Leading to the Induction of rDCs by Stromal Cells

It is clear that in several tissue contexts stromal cells can induce rDCs, but direct evidence as to whether this process is regulated by infection or inflammation is more limited. One exception is experimental visceral leishmaniasis (EVL), a chronic infection caused by the intracellular parasite *L. donovani* (Kaye et al., 2004). Splenic stromal cells from mice infected with *L. donovani* have an enhanced capacity to direct hematopoietic progenitors toward a rDC phenotype *in vitro* (Svensson et al., 2004), a process at least in part dependent upon infection-modulated levels of the chemokine CCL8 (Nguyen Hoang et al., 2010). The precise mechanisms by which infection itself enhances the capacity for stromal cells to support rDC induction during chronic inflammation are not known, but as stromal cells are targets of *Leishmania* infection (Bogdan et al., 2000) it is feasible that direct parasite modulation of stromal cell function may represent a strategy for manipulating host defense mechanisms in favor of the invading pathogen (Svensson and Kaye, 2006). Whether similar alterations in stromal cells occur during other parasitic infections with an abundance of CD11c^lo^rDCs (Li et al., 2011; Smith et al., 2011), and whether rDCs are associated with chronic infection by viral or bacterial pathogens will require further investigation.

As stromal cells from organs considered mucosal (Li et al., 2008; Huang et al., 2009) or with specialized properties related to tolerance induction (Xia et al., 2008) have been reported to induce rDCs, it is possible that this is a generalized feature of stromal populations from these sites. It is not yet known whether stromal cells from other mucosal organs, such as the skin or intestine, are specialized for the induction of rDCs.

## Mechanisms of rDC Induction by Stromal Cells

Unlike the relatively conserved phenotypic and functional characteristics of rDCs, there are multiple reported mechanisms by which stromal cells induce these cells. Physical contact between splenic stromal cells and mature cDCs has been shown to be required for their polarization toward a rDC phenotype, in a process also dependent on fibronectin (Zhang et al., 2004). However, kidney stromal cells can induce rDCs by a process that does not require cell-cell contact (Huang et al., 2009), indicating heterogeneity in the mechanisms underlying stromal cell-induced rDC differentiation. This is perhaps not surprising given the heterogeneity in stromal cell populations themselves, as shown clearly by recent transcriptional analysis of stromal cell subsets within lymphoid tissue (Malhotra et al., 2012).

Diverse stromal cell-derived products such as IL-6 (Huang et al., 2009), CCL8 and CXCL12 (Nguyen Hoang et al., 2010), TGFβ (Li et al., 2008), and M-CSF (Xia et al., 2008) have been reported to impact upon the stromal cell induction of rDCs, but it is unclear as to whether redundancy exists in this system. In addition to rDCs being producers of PGE_2_ and IL-10, the release of these mediators by stromal cells has also been implicated in their generation, with splenic endothelial-produced IL-10 (Tang et al., 2006) and tumor stroma-derived PGE_2_ (Liu et al., 2009) playing a role in the induction of rDC populations.

A potent immunoregulatory circuit endowing human DCs with IL-27-dependent regulatory potential was recently described (Ilarregui et al., 2009), relying on the carbohydrate-binding protein Galectin-1. As Galectin-1 is expressed by stromal cells (Jung et al., 2007), this raises the intriguing possibility that stromal populations are also capable of delivering signals that skew conventional human DCs toward regulatory capacity, in addition to the differentiation of specific rDC populations.

## Future Questions

As evidenced by this short review, there is still much to be revealed regarding the precise mechanisms by which stromal cells induce the differentiation and/or expansion of rDCs (Figure 1). Indeed there are large gaps in our knowledge regarding the precise identity of rDCs, whether subsets of these cells exist *in vivo* and the lineage relationship of rDCs to conventional DC populations. Transcriptional and epigenetic profiling of rDCs from a multitude of tissue sites and disease states will allow for these questions to begin to be addressed. As many of the mediators previously reported to be important for rDC generation are produced widely within the immune system, it is important to determine how they contribute to the generation of rDCs within a defined tissue microenvironment. As it is likely that a multitude of synergistic signals underlie the induction of rDCs by stromal cells, revealing the extent of redundancy in this system will be key in finding pathways essential for rDC induction by stromal cells. In addition, extending our knowledge of the processes by which pathogens, vaccination, or chronic inflammation can modulate stromal cell function, and thus favor or suppress rDC induction *in vivo* will be crucial when considering therapeutic strategies aimed at manipulating rDC abundance or function.

Furthermore, a more detailed analysis of the stromal microenvironment that supports rDC generation *in vitro* will provide clues as to novel mechanisms responsible for their *in vivo* induction. This type of analysis has already been performed for splenic stromal cell populations capable of inducing immature DC populations *in vitro* (Despars et al., 2008), identifying gene signatures associated with this functional capacity. Extending this approach to rDC induction will likely reveal much useful data in this area.

More fundamentally, it is essential that we gain a much deeper understanding of the stromal cell populations capable of rDC induction *in vivo*. Even within the same organ it would appear that diverse stromal populations such as fibroblasts (Svensson et al., 2004) and endothelial cells (Tang et al., 2006) are capable of rDC induction. Clarity on whether distinct differences in stromal cells both within and between organs results in a differing capacity for rDC induction, or alternative mechanisms by which induction occurs, will likely reveal much about the biological processes required for stromal cells to induce rDC populations. Key to this may be identification of the tissue specific niches for rDC development (as this will define the local stromal cell population) coupled to transcriptional and/or proteomic analysis of the stromal cells at such sites. In particular the application of advanced imaging techniques such as intravital microscopy or whole mount histology could be applied to identifying stromal niches for rDC generation *in situ*, allowing for the visualization of the distinct microenvironments that facilitate the induction of these cells. However these approaches will necessitate the development of specific tools for the identification of rDCs *in vivo*. Such experiments are also likely to challenge the conclusions drawn from conventional experimental approaches.

To bring clarity to the field, the following approaches should therefore be taken to address the major outstanding research questions regarding stromal cell-induced rDCs:

– Transcriptional and epigenetic analysis of stromal-induced rDCs from distinct tissues to determine whether subsets exist and their relationship to cDCs.
– Imaging approaches to visualize the stromal cell-rDC niche *in vivo*, which will require the development of new tools for rDC identification.
– Transcriptional and epigenetic analysis of stromal cells capable of rDC induction, specifically comparing these profiles to those of stromal cells from distinct tissues and/or disease states.

Extending our knowledge of both rDCs and the stromal cells that induce them may allow for the potential therapeutic benefits of this immunoregulatory axis to be realized.

## Harnessing Stromal Cell Niches for Therapeutic Immune Regulation

With patient-specific pro-inflammatory DC infusions effective in phase III trials for prostate cancer (Kantoff et al., 2010), the use of myeloid cell populations to modulate immune responses in humans is rapidly becoming a clinical reality. Applying this rationale to the design of alternative therapeutic myeloid cell infusions aimed instead at repressing immune responses is already under close scrutiny (Kalantari et al., 2011; Lutz, 2012), with progress in this area likely soon. As stromal cell-induced rDCs are potent negative regulators of inflammation and improve outcome in pre-clinical models of hepatic and pulmonary insult (Li et al., 2008; Xia et al., 2008), such cell populations – once characterized fully in humans – would provide a promising candidate for therapeutic infusion.

Although clearly feasible, the *ex vivo* expansion of myeloid cell populations for infusion has disadvantages. Instead, harnessing the capacity for stromal cells to regulate rDC induction by specifically targeting them for functional modulation *in vivo* could provide a method to enhance rDC induction for the amelioration of autoimmune or inflammatory disease, or conversely repress rDC induction during the immunosuppression associated with chronic infection and cancer. Stromal targeting approaches have already attracted much interest in the oncology field (Engels et al., 2012). Experimental therapeutics have included the targeting of potent cytotoxic agents to tumor stroma by conjugation to a collagen IV-specific monoclonal antibody (Yasunaga et al., 2011), specific enzymatic degradation of tumor stroma to enhance stromal remodeling (Provenzano et al., 2012), and therapeutic ablation of cancer stromal cells in murine tumor models (Kraman et al., 2010), but stromal targeting approaches are not usually considered in other therapeutic contexts.

By expanding our knowledge regarding the detailed mechanisms underlying stromal cell – regulatory dendritic cell interactions, we may advance one step closer to the ultimate goal of subtly manipulating immune function by targeting stromal cells within an inflammatory microenvironment.

## Conflict of Interest Statement

The authors declare that the research was conducted in the absence of any commercial or financial relationships that could be construed as a potential conflict of interest.
